# Endovascular Treatment of Traumatic Vertebral Artery Pseudoaneurysm

**DOI:** 10.7759/cureus.79271

**Published:** 2025-02-19

**Authors:** Noora Aljalahma, Fatema Husain, Amr Ashour, Martin Maresch

**Affiliations:** 1 Surgery, Bahrain Defence Force Royal Medical Services, Military Hospital, Riffa, BHR; 2 General Practice, Salmaniya Medical Complex, Manama, BHR; 3 Vascular Surgery, Bahrain Defence Force Royal Medical Services, Military Hospital, Riffa, BHR

**Keywords:** angiogram, computed tomography, covered stent, pseudoaneurysm, stab wound, vertebral artery

## Abstract

Vertebral artery pseudoaneurysm usually occurs following penetrating trauma and is associated with a high mortality rate. Computed tomographic angiography (CTA) is usually the diagnostic method of choice. We present a case of pseudoaneurysm of the left vertebral artery caused by a stab wound to the left neck, which was treated with an endovascular approach with a covered stent.

## Introduction

Traumatic extracranial vertebral artery pseudoaneurysm is a rare condition, accounting for less than 1% of all aneurysms [1]. Majidi et al. [2] reported that the incidence of vertebral artery dissection was 0.01% of patients with trauma in the head and neck. Furthermore, traumatic pseudoaneurysms are associated with a high mortality rate and can result in hematoma, arteriovenous fistula, or complete occlusion. Additionally, their rates of morbidity are high as patients are at risk of having a stroke [2]. Here, we report on a rare case of post-traumatic pseudoaneurysm arising from the first segment of the left vertebral artery (V1).

## Case presentation

**Figure 1:**
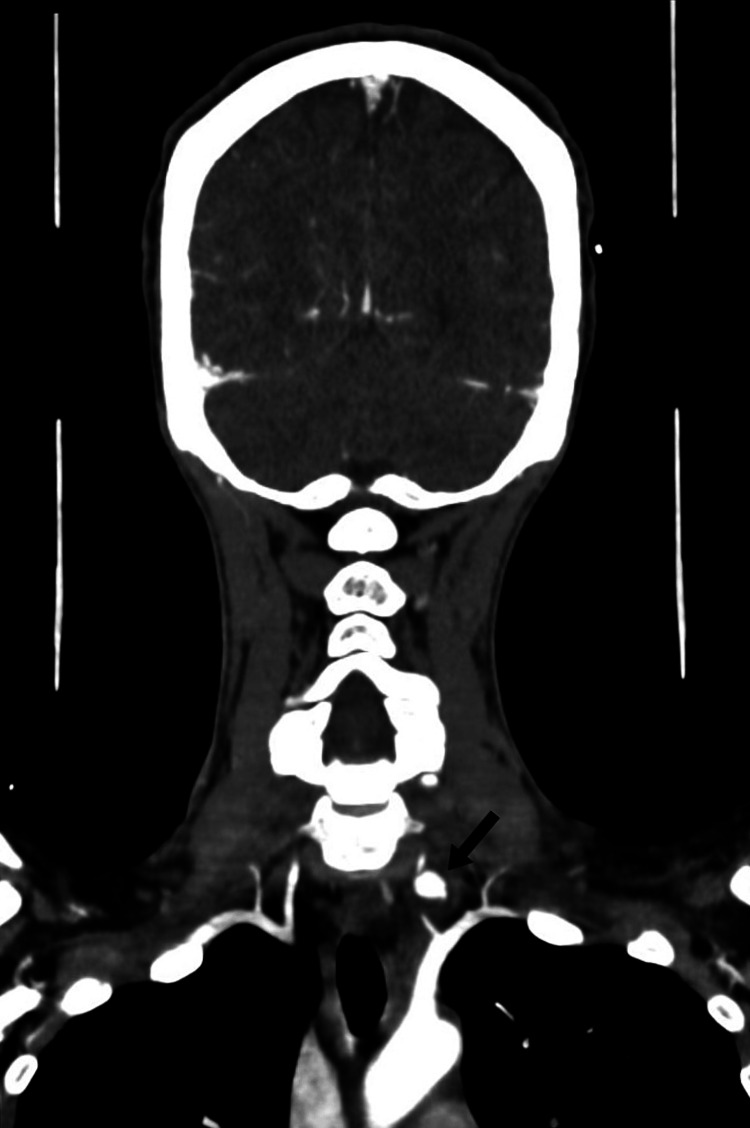
Preoperative computed tomographic angiography (coronal cut) demonstrating extravasation from the first segment of the left vertebral artery with pseudoaneurysm formation (arrow) CTA: computed tomography angiography

A 38-year-old female presented to the emergency department after a stab wound to the left side of her neck, which measured approximately 2 x 2 cm. On admission, the patient was hemodynamically stable, and the Advanced Trauma Life Support (ATLS) protocol was immediately applied to the patient. The patient had swelling in the left-sided neck and a non-expanding and non-pulsatile hematoma. Computed tomographic angiography (CTA) of the neck and brain was done immediately, which showed slight extravasation from the left (V1) with an intact circle of Willis (Figure 1).

**Figure 2:**
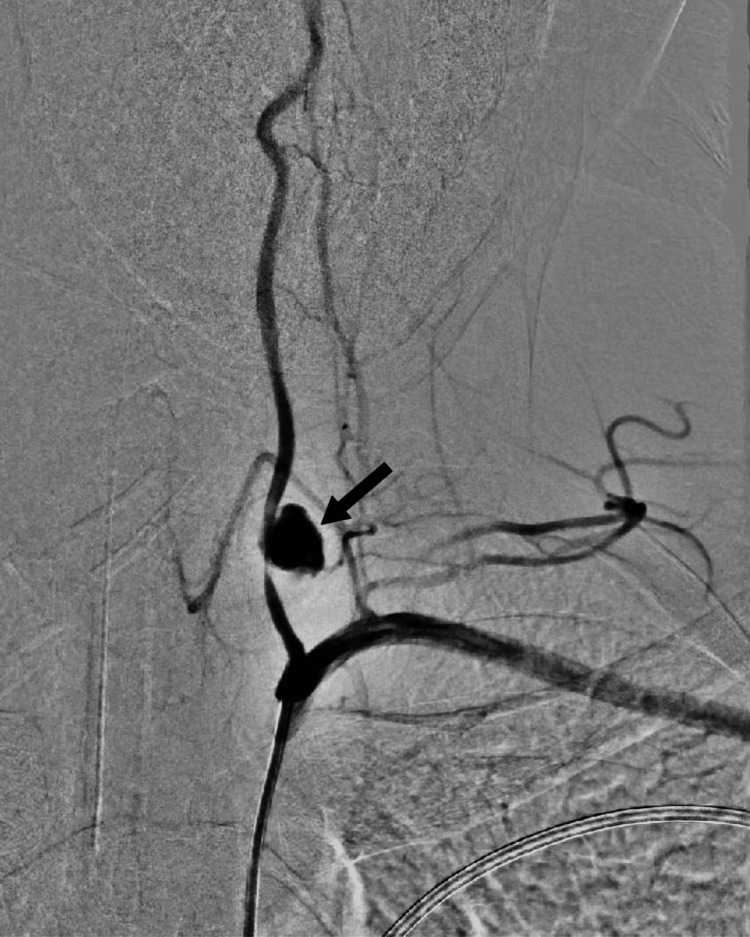
Intraoperative arteriogram demonstrating pseudoaneurysm from the first segment of the vertebral artery (arrow)

Following consultations with vascular surgery, interventional radiology, and neurovascular surgery teams, the patient was taken immediately to a hybrid operating theater. The patient underwent general anesthesia, and an intravenous bolus of 5000 international units (IU) of heparin was given. Additionally, a diagnostic angiogram was done through right femoral artery access, which showed pseudoaneurysm at left (V1) with extravasation with an intact circle of Willis (Figure 2).

**Figure 3:**
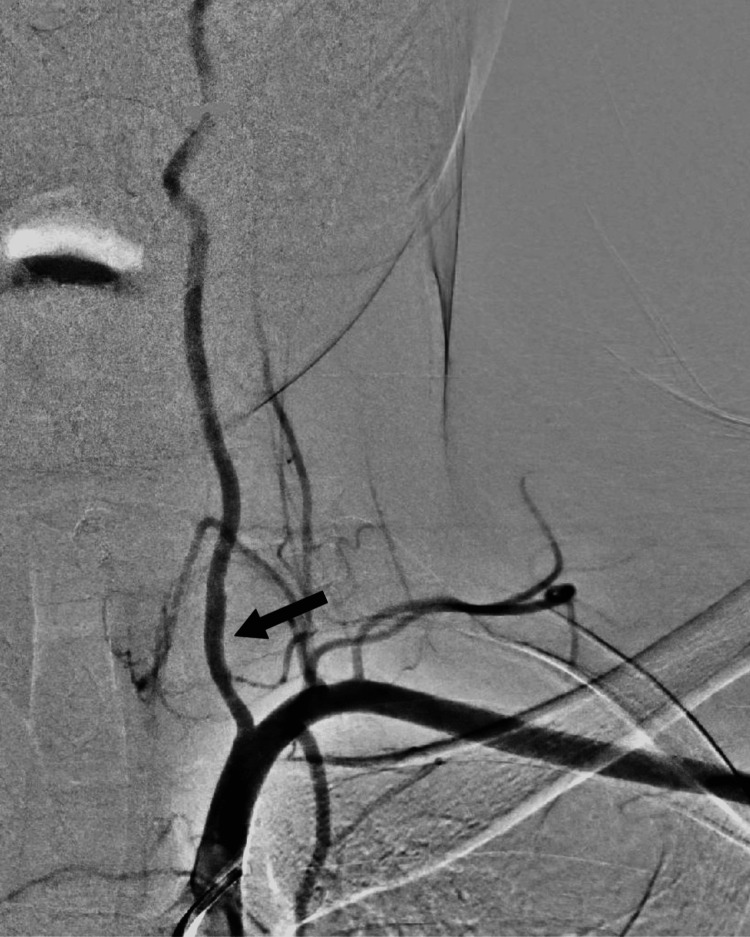
Intraoperative arteriogram showing pseudoaneurysm from the first segment of the vertebral artery (arrow)

The left (V1) segment was crossed with 014 hydrophilic wire without complications. A 3 mm x 20 mm covered stent was deployed successfully with no local complications. The completion of the angiogram showed complete exclusion of pseudoaneurysm and patent vertebrobasilar circulation (Figure 3). The patient was kept in the ward for observation for 48 hours and then discharged safely on double antiplatelet therapy.

**Figure 4:**
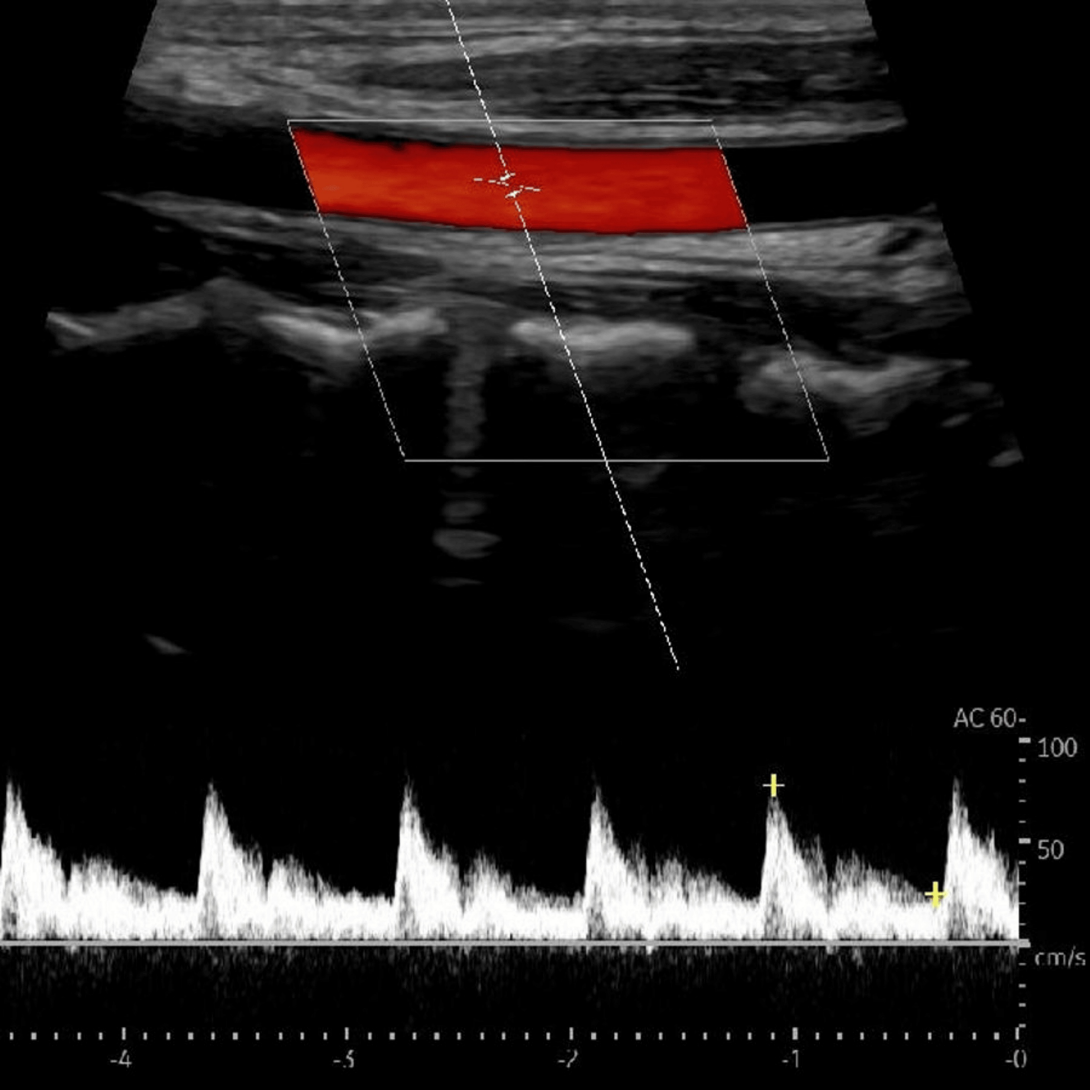
Arterial ultrasound duplex showing a patent vertebral artery stent

**Figure 5:**
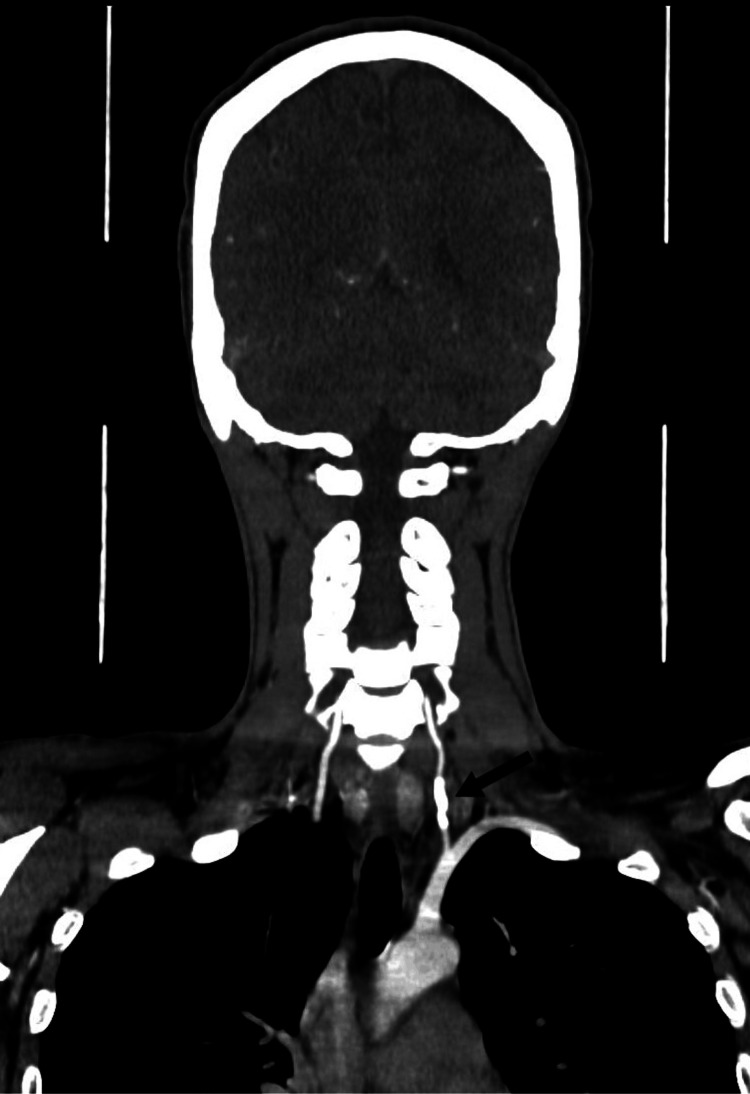
Computed tomography angiographic of the head, neck, and brain showing the patent stent in the left vertebral artery (arrow)

On an outpatient follow-up, the patient was stable and found to have ptosis. Thus, was referred to neurology for assessment. After one month, a follow-up ultrasound duplex of neck vessels was performed and showed a patent stent (Figure 4). Also, CTA of the head, neck, and brain was done and showed a patent stent in the left (V1) (Figure 5).

## Discussion

Vertebral artery pseudoaneurysms are rare in presentation; they may occur following a trauma injury, radiation, invasion of a tumor, and an infection. Furthermore, they must be treated because if they are left without intervention, they may lead to complications like bleeding, occluded vessels, expansion of the vessel, and distal thromboembolism [3].

CTA is widely available and, in the absence of contraindications, is usually the first step in the diagnosis and evaluation of suspected vascular trauma. However, DSA may be warranted in cases where artifact due to the presence of metallic foreign bodies interferes with the interpretation of CT-based studies [4]. To illustrate, our patient underwent CTA, which indicated slight extravasation from the left (V1) with an intact circle of Willis.

Certainly, when it comes to management, it depends on the individual needs. Surgery is preferred when the pseudoaneurysm is more proximal, and if it is in the third or fourth segments, it is safer to go with the percutaneous approach [5]. If pseudoaneurysms were accessible, they used to be treated surgically, but if it is not, patients were given anti-coagulations and antiplatelets, which did not show excellent outcomes. Nowadays, doctors are treating pseudoaneurysms with covered stents, which is considered part of endovascular management, and it turns out they are safe and effective [3]. Additionally, the advantage of using a covered stent is that it will seal the lesion, eliminate the pseudoaneurysm, and save the vertebral artery. However, pseudoaneurysms occurring in the third or fourth segment are more challenging to manage [4]. In our patient, a covered stent was used, and an angiogram showed elimination of the pseudoaneurysm as well as patent vertebrobasilar circulation, and, thankfully, no complications were reported.

Also, the contralateral vertebral artery, with incomplete treatment, can be accessed via up and over retrograde. Indeed, this technique is beneficial in patients with polytrauma who are unstable. Furthermore, coil embolization is a safe option when there is patency in the contralateral vertebral artery. However, if it is occluded, then it is better to go with the bare metal stenting with embolization by stent fenestrations or covered stent. According to a study done in 2022, a number of 18 penetrating cases of vertebral artery pseudoaneurysm were treated by coil embolization, balloon occlusion, and stent placement. The result was successful, with no complications [6].

## Conclusions

Vertebral artery pseudoaneurysms are considered rare; they can appear due to multiple causes, either due to invasion of a tumor or an infection or due to trauma (penetrating), like what happened with the patient presented in this case report. The optimal method of diagnosis is CTA when it comes to the diagnosis of vascular lesions. Additionally, it is considered an emergency due to the high risk of thrombosis or rupture, and covered stents have shown excellent results in treating injuries related to vertebral arteries. This case report demonstrates how noninvasive endovascular therapies may be employed in the definitive management of penetrating vertebral artery injuries.
